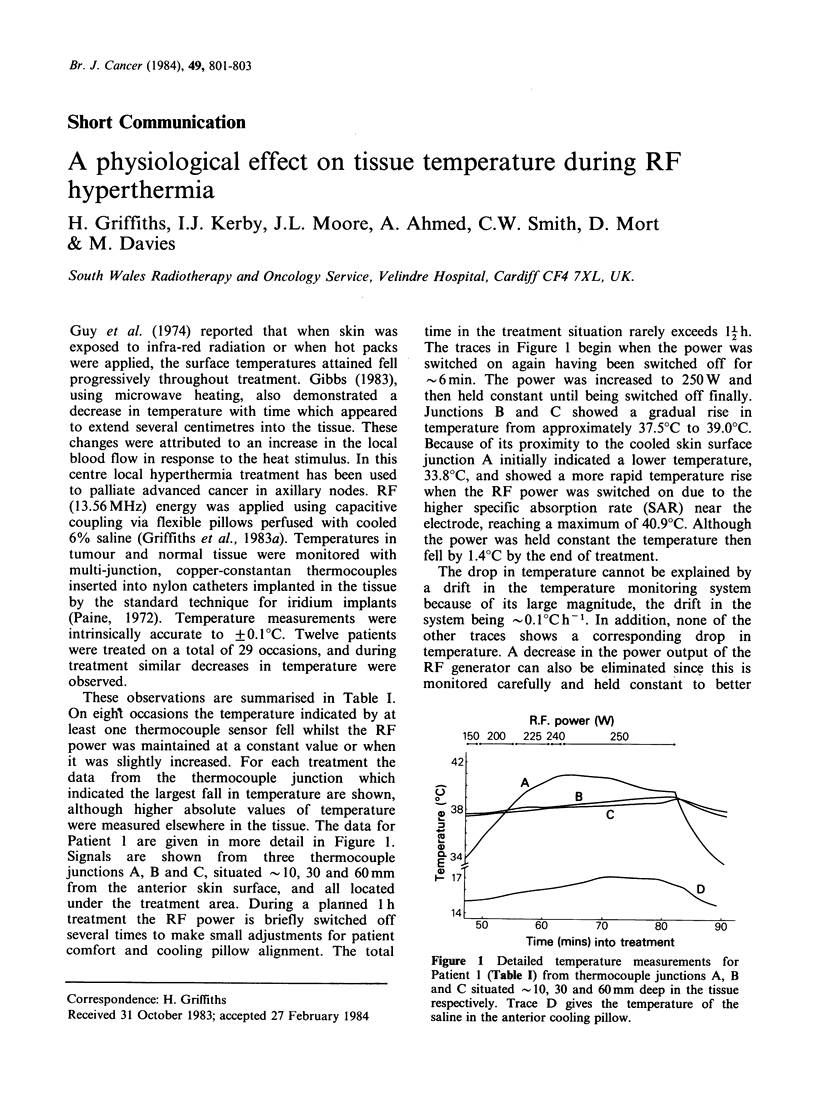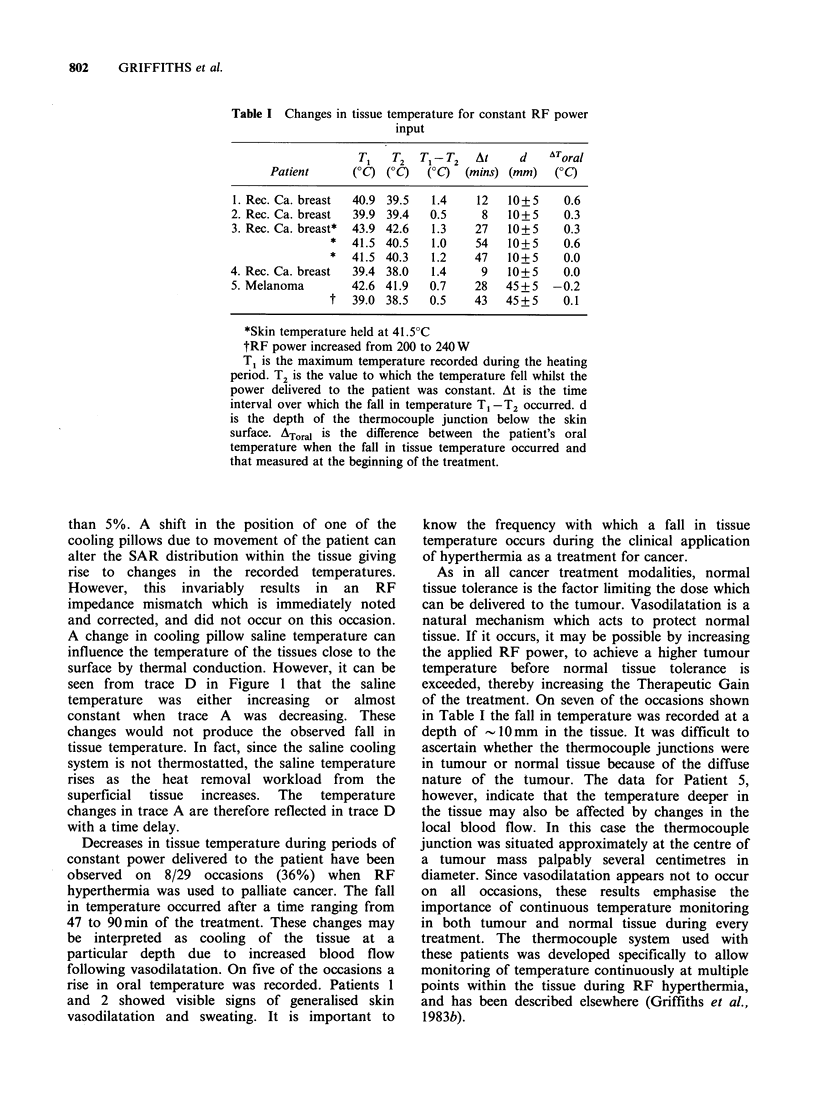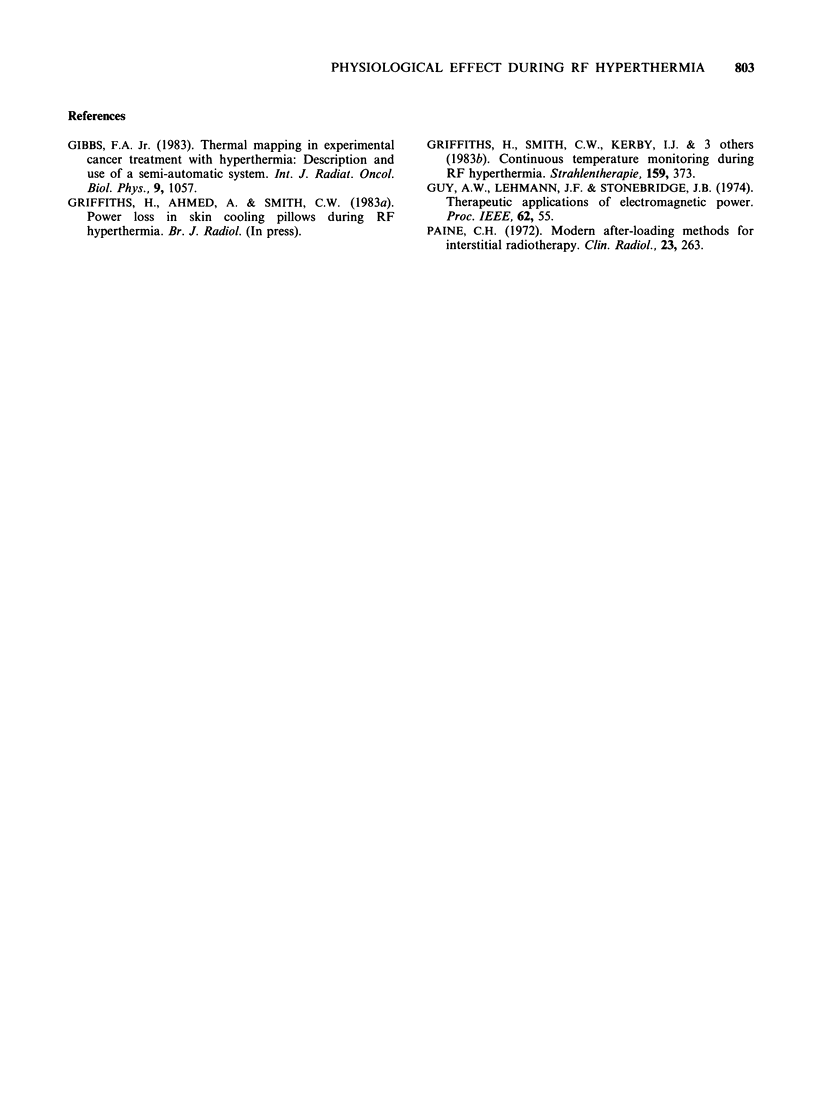# A physiological effect on tissue temperature during RF hyperthermia.

**DOI:** 10.1038/bjc.1984.124

**Published:** 1984-06

**Authors:** H. Griffiths, I. J. Kerby, J. L. Moore, A. Ahmed, C. W. Smith, D. Mort, M. Davies


*Br. J. Cancer* (1984), **49**, 801-803

### Short Communication

### A physiological effect on tissue temperature during RF hyperthermia

H. Griffiths, I.J. Kerby, J.L. Moore, A. Ahmed, C.W. Smith, D. Mort & M. Davies

*South Wales Radiotherapy and Oncology Service, Velindre Hospital, Cardiff CF4 7XL, UK.*

Guy *et al.* (1974) reported that when skin was exposed to infra-red radiation or when hot packs were applied, the surface temperatures attained fell progressively throughout treatment. Gibbs (1983), using microwave heating, also demonstrated a decrease in temperature with time which appeared to extend several centimetres into the tissue. These changes were attributed to an increase in the local blood flow in response to the heat stimulus. In this centre local hyperthermia treatment has been used to palliate advanced cancer in axillary nodes. RF (13.56 MHz) energy was applied using capacitive coupling via flexible pillows perfused with cooled 6% saline (Griffiths *et al.*, 1983*a*). Temperatures in tumour and normal tissue were monitored with multi-junction, copper-constantan thermocouples inserted into nylon catheters implanted in the tissue by the standard technique for iridium implants (Paine, 1972). Temperature measurements were intrinsically accurate to $\pm 0.1$°C. Twelve patients were treated on a total of 29 occasions, and during treatment similar decreases in temperature were observed.

These observations are summarised in Table I. On eight occasions the temperature indicated by at least one thermocouple sensor fell whilst the RF power was maintained at a constant value or when it was slightly increased. For each treatment the data from the thermocouple junction which indicated the largest fall in temperature are shown, although higher absolute values of temperature were measured elsewhere in the tissue. The data for Patient 1 are given in more detail in Figure 1. Signals are shown from three thermocouple junctions A, B and C, situated $\sim$10, 30 and 60 mm from the anterior skin surface, and all located under the treatment area. During a planned 1 h treatment the RF power is briefly switched off several times to make small adjustments for patient comfort and cooling pillow alignment. The total time in the treatment situation rarely exceeds $1\frac{1}{2}$ h. The traces in Figure 1 begin when the power was switched on again having been switched off for $\sim$6 min. The power was increased to 250 W and then held constant until being switched off finally. Junctions B and C showed a gradual rise in temperature from approximately 37.5°C to 39.0°C. Because of its proximity to the cooled skin surface junction A initially indicated a lower temperature, 33.8°C, and showed a more rapid temperature rise when the RF power was switched on due to the higher specific absorption rate (SAR) near the electrode, reaching a maximum of 40.9°C. Although the power was held constant the temperature then fell by 1.4°C by the end of treatment.

The drop in temperature cannot be explained by a drift in the temperature monitoring system because of its large magnitude, the drift in the system being $\sim 0.1$°C h$^{-1}$. In addition, none of the other traces shows a corresponding drop in temperature. A decrease in the power output of the RF generator can also be eliminated since this is monitored carefully and held constant to better

**Figure 1** Detailed temperature measurements for Patient 1 (**Table I**) from thermocouple junctions A, B and C situated $\sim$10, 30 and 60 mm deep in the tissue respectively. Trace D gives the temperature of the saline in the anterior cooling pillow.

Correspondence: H. Griffiths

Received 31 October 1983; accepted 27 February 1984

**Table I** Changes in tissue temperature for constant RF power input

| *Patient* | $T_1$ (°C) | $T_2$ (°C) | $T_1-T_2$ (°C) | $\Delta t$ (*mins*) | *d* (*mm*) | $^{\Delta T}oral$ (°C) |
|---|---|---|---|---|---|---|
| 1. Rec. Ca. breast | 40.9 | 39.5 | 1.4 | 12 | 10±5 | 0.6 |
| 2. Rec. Ca. breast | 39.9 | 39.4 | 0.5 | 8 | 10±5 | 0.3 |
| 3. Rec. Ca. breast* | 43.9 | 42.6 | 1.3 | 27 | 10±5 | 0.3 |
| * | 41.5 | 40.5 | 1.0 | 54 | 10±5 | 0.6 |
| * | 41.5 | 40.3 | 1.2 | 47 | 10±5 | 0.0 |
| 4. Rec. Ca. breast | 39.4 | 38.0 | 1.4 | 9 | 10±5 | 0.0 |
| 5. Melanoma | 42.6 | 41.9 | 0.7 | 28 | 45±5 | −0.2 |
| † | 39.0 | 38.5 | 0.5 | 43 | 45±5 | 0.1 |

*Skin temperature held at 41.5°C
†RF power increased from 200 to 240 W
$T_1$ is the maximum temperature recorded during the heating period. $T_2$ is the value to which the temperature fell whilst the power delivered to the patient was constant. Δt is the time interval over which the fall in temperature $T_1-T_2$ occurred. d is the depth of the thermocouple junction below the skin surface. $\Delta_{Toral}$ is the difference between the patient's oral temperature when the fall in tissue temperature occurred and that measured at the beginning of the treatment.

than 5%. A shift in the position of one of the cooling pillows due to movement of the patient can alter the SAR distribution within the tissue giving rise to changes in the recorded temperatures. However, this invariably results in an RF impedance mismatch which is immediately noted and corrected, and did not occur on this occasion. A change in cooling pillow saline temperature can influence the temperature of the tissues close to the surface by thermal conduction. However, it can be seen from trace D in Figure 1 that the saline temperature was either increasing or almost constant when trace A was decreasing. These changes would not produce the observed fall in tissue temperature. In fact, since the saline cooling system is not thermostatted, the saline temperature rises as the heat removal workload from the superficial tissue increases. The temperature changes in trace A are therefore reflected in trace D with a time delay.

Decreases in tissue temperature during periods of constant power delivered to the patient have been observed on 8/29 occasions (36%) when RF hyperthermia was used to palliate cancer. The fall in temperature occurred after a time ranging from 47 to 90 min of the treatment. These changes may be interpreted as cooling of the tissue at a particular depth due to increased blood flow following vasodilatation. On five of the occasions a rise in oral temperature was recorded. Patients 1 and 2 showed visible signs of generalised skin vasodilatation and sweating. It is important to know the frequency with which a fall in tissue temperature occurs during the clinical application of hyperthermia as a treatment for cancer.

As in all cancer treatment modalities, normal tissue tolerance is the factor limiting the dose which can be delivered to the tumour. Vasodilatation is a natural mechanism which acts to protect normal tissue. If it occurs, it may be possible by increasing the applied RF power, to achieve a higher tumour temperature before normal tissue tolerance is exceeded, thereby increasing the Therapeutic Gain of the treatment. On seven of the occasions shown in Table I the fall in temperature was recorded at a depth of ~10 mm in the tissue. It was difficult to ascertain whether the thermocouple junctions were in tumour or normal tissue because of the diffuse nature of the tumour. The data for Patient 5, however, indicate that the temperature deeper in the tissue may also be affected by changes in the local blood flow. In this case the thermocouple junction was situated approximately at the centre of a tumour mass palpably several centimetres in diameter. Since vasodilatation appears not to occur on all occasions, these results emphasise the importance of continuous temperature monitoring in both tumour and normal tissue during every treatment. The thermocouple system used with these patients was developed specifically to allow monitoring of temperature continuously at multiple points within the tissue during RF hyperthermia, and has been described elsewhere (Griffiths *et al.*, 1983*b*).

### References

GIBBS, F.A. Jr. (1983). Thermal mapping in experimental cancer treatment with hyperthermia: Description and use of a semi-automatic system. *Int. J. Radiat. Oncol. Biol. Phys.*, **9**, 1057.

GRIFFITHS, H., AHMED, A. & SMITH, C.W. (1983*a*). Power loss in skin cooling pillows during RF hyperthermia. *Br. J. Radiol.* (In press).

GRIFFITHS, H., SMITH, C.W., KERBY, I.J. & 3 others (1983*b*). Continuous temperature monitoring during RF hyperthermia. *Strahlentherapie*, **159**, 373.

GUY, A.W., LEHMANN, J.F. & STONEBRIDGE, J.B. (1974). Therapeutic applications of electromagnetic power. *Proc. IEEE*, **62**, 55.

PAINE, C.H. (1972). Modern after-loading methods for interstitial radiotherapy. *Clin. Radiol.*, **23**, 263.